# Metasurface designed with quantitative field distributions

**DOI:** 10.1038/s41377-023-01155-w

**Published:** 2023-05-09

**Authors:** Shuai Wang, Yuanmu Yang

**Affiliations:** State Key Laboratory for Precision Measurement Technology and Instruments, Department of Precision Instrument, Tsinghua University, Beijing, 100084 China

**Keywords:** Metamaterials, Imaging and sensing

## Abstract

A new method for designing metasurfaces has been proposed and demonstrated, which allows for the generation of precise quantitative field distributions. This unique approach involves combining a tandem neural network with an iterative algorithm to optimize the metasurface design, enabling accurate control over the intensity and polarization of the resulting field. This strategy is both efficient and robust and has the potential to accelerate the development of metasurface devices with complex functionalities.

Metasurface, a new class of planar diffractive elements composed of an array of subwavelength meta-atoms, has been demonstrated to be extremely versatile in manipulating the vectorial electromagnetic fields, including the wavefront^1–4^ and polarization^5–8^. The conventional strategy for designing a metasurface is based on the independent assembly of a series of meta-atoms with different shapes and orientations. Such an approach requires constructing a map connecting the required electromagnetic response with the meta-atom geometry by sweeping over a wide structural parameter space, resulting in time-consuming simulations.

Artificial neural networks (ANNs) are complex systems with numerous interconnections between multiple layers. It can be trained to establish the relationship between the electromagnetic response and the geometry of the meta-atom in an efficient manner^9,10^. ANNs can be broadly divided into two categories, forward neural networks and inverse neural networks, based on their function in the metasurface design. Forward neural networks predict the electromagnetic response of input meta-atoms with varying materials and shapes^11^, replacing time-consuming full-wave electromagnetic simulations. Inverse neural networks are trained to directly identify the metasurface design with a targeted electromagnetic response^12^. Despite much recent progress, it remains challenging to identify ANN-assisted metasurface design approaches for the automated generation of an electromagnetic field with precisely determined, quantitative field distributions. For instance, for polarization-splitting metalens, the intensity ratio between two orthogonal polarization states needs to be manually controlled.

In a recent work^13^ published in Light: Advanced Manufacturing, Prof. Xiaofei Zang, Prof. Yiming Zhu, and collaborators from the University of Shanghai for Science and Technology and Tongji University have proposed a new metasurface design approach, which combines a bidirectional deep neural network and an iterative algorithm, to generate a more quantitative field distribution. In the first step, the bidirectional deep neural network consisting of a predicting neural network and a pre-trained forward neural network is designed to generate a qualitative field distribution. Subsequently, to enable precise and automated manipulation of the spatial distribution of the vectorial electromagnetic field, including the amplitude, phase, and polarization, an iterative algorithm is added to the tandem neural network to predict metasurface devices with more precisely controlled field distributions, as schematically shown in Fig. 1.

**Fig. 1:**
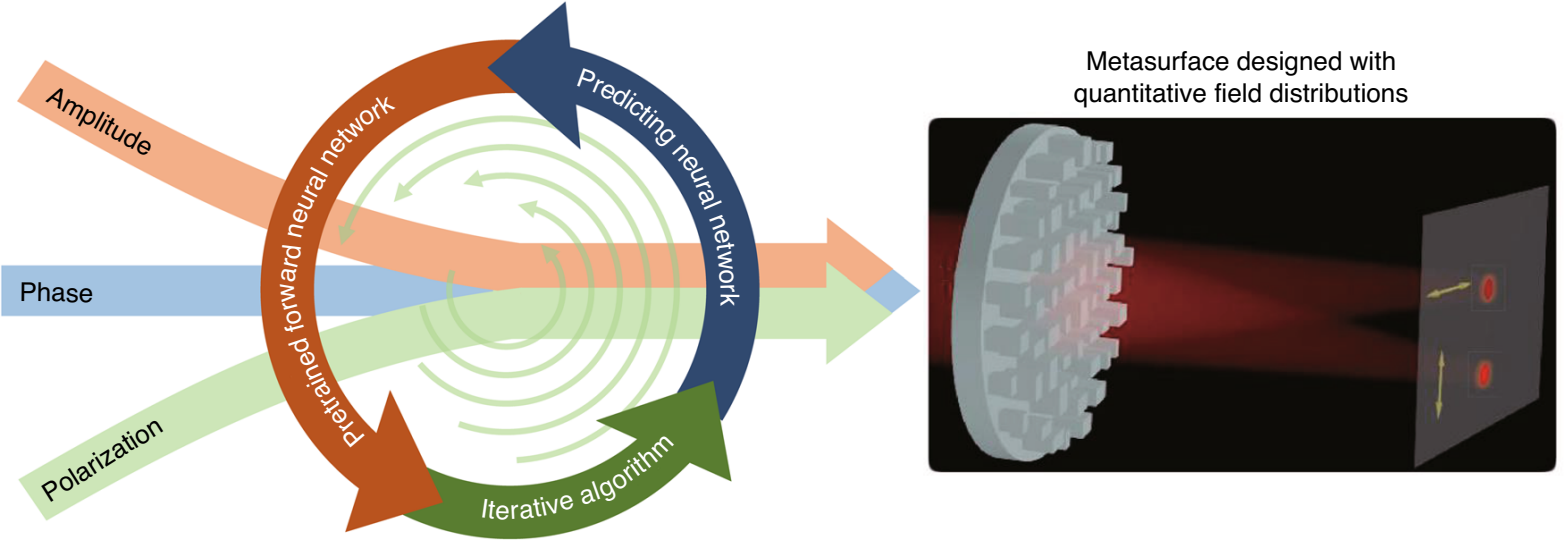
The metasurface with quantitative field distribution predicted by tandem neural networks and iterative algorithms. Combining a bidirectional deep neural network and an iterative algorithm to design a metasurface with a quantitative field distribution. The input parameters include amplitude, phase, and polarization, as shown on the left panel. The schematic on the right panel illustrates the predicted metasurface for generating focal spots with customized polarization and intensity ratio

To validate the proposed approach, a series of metasurface devices have been designed, including metalenses that generate two foci with intensity ratios varying from 1:1 to 0.6:1 and with either identical or orthogonal polarization states and vortex generators for generating position- and polarization-dependent converged vortices. The designed metasurfaces have been fabricated and experimentally measured in the terahertz frequency regime, showing close agreement with the numerical predictions.

The method proposed in the presented work further extended the capability of ANNs to identify proper metasurface designs with stringently tailored responses in a straightforward and efficient manner. It provides a platform for globally optimizing complex vectorial electromagnetic responses of metasurfaces, which may be useful for applications including parallel laser direct writing, polarization imaging, and spatially multiplexed optical communication.

## References

[1] Pan MY (2022). Dielectric metalens for miniaturized imaging systems: progress and challenges. Light Sci. Appl..

[2] Sun SL (2012). High-efficiency broadband anomalous reflection by gradient meta-surfaces. Nano Lett..

[3] Li LL (2022). Intelligent metasurfaces: control, communication and computing. eLight.

[4] Shen ZC (2023). Monocular metasurface camera for passive single-shot 4D imaging. Nat. Commun..

[5] Yang YM (2014). Dielectric meta-reflectarray for broadband linear polarization conversion and optical vortex generation. Nano Lett..

[6] Wang S (2021). Arbitrary polarization conversion dichroism metasurfaces for all-in-one full Poincare sphere polarizers. Light Sci. Appl..

[7] Zhao F (2021). Metalens-assisted system for underwater imaging. Laser Photonics Rev..

[8] Ni YB (2022). Computational spectropolarimetry with a tunable liquid crystal metasurface. eLight.

[9] Ma W (2021). Deep learning for the design of photonic structures. Nat. Photonics.

[10] Chen MK (2022). Artificial intelligence in meta-optics. Chem. Rev..

[11] Qu YR (2019). Migrating knowledge between physical scenarios based on artificial neural networks. ACS Photonics.

[12] Wang FL (2022). Visible achromatic metalens design based on artificial neural network. Adv. Optical Mater..

[13] Zhu Y (2023). Metasurfaces designed by a bidirectional deep neural network and iterative algorithm for generating quantitative field distributions. Light Adv. Manuf..

